# Evaluating the Good Ontology Design Guideline (GoodOD) with the Ontology Quality Requirements and Evaluation Method and Metrics (OQuaRE)

**DOI:** 10.1371/journal.pone.0104463

**Published:** 2014-08-22

**Authors:** Astrid Duque-Ramos, Martin Boeker, Ludger Jansen, Stefan Schulz, Miguela Iniesta, Jesualdo Tomás Fernández-Breis

**Affiliations:** 1 Departamento de Informática y Sistemas, Facultad de Informática, Universidad de Murcia, Murcia, Spain; 2 Department of Medical Biometry and Medical Informatics, University of Freiburg, Freiburg, Germany; 3 Department of Philosophy, University of Muenster, Muenster, Germany; 4 Institute of Medical Informatics, Statistics and Documentation Medical University of Graz, Graz, Austria; 5 Departamento de Estadística e Investigación Operativa, Universidad de Murcia, Murcia, Spain; University of Aberystwyth, United Kingdom

## Abstract

**Objective:**

To (1) evaluate the GoodOD guideline for ontology development by applying the OQuaRE evaluation method and metrics to the ontology artefacts that were produced by students in a randomized controlled trial, and (2) informally compare the OQuaRE evaluation method with gold standard and competency questions based evaluation methods, respectively.

**Background:**

In the last decades many methods for ontology construction and ontology evaluation have been proposed. However, none of them has become a standard and there is no empirical evidence of comparative evaluation of such methods. This paper brings together GoodOD and OQuaRE. GoodOD is a guideline for developing robust ontologies. It was previously evaluated in a randomized controlled trial employing metrics based on gold standard ontologies and competency questions as outcome parameters. OQuaRE is a method for ontology quality evaluation which adapts the SQuaRE standard for software product quality to ontologies and has been successfully used for evaluating the quality of ontologies.

**Methods:**

In this paper, we evaluate the effect of training in ontology construction based on the GoodOD guideline within the OQuaRE quality evaluation framework and compare the results with those obtained for the previous studies based on the same data.

**Results:**

Our results show a significant effect of the GoodOD training over developed ontologies by topics: (a) a highly significant effect was detected in three topics from the analysis of the ontologies of untrained and trained students; (b) both positive and negative training effects with respect to the gold standard were found for five topics.

**Conclusion:**

The GoodOD guideline had a significant effect over the quality of the ontologies developed. Our results show that GoodOD ontologies can be effectively evaluated using OQuaRE and that OQuaRE is able to provide additional useful information about the quality of the GoodOD ontologies.

## Introduction

In recent years, ontologies have been successfully applied in different domains including e-commerce [1], biomedicine [2], [3], e-learning [4] or geospatial data [5]. Their success is based mainly on the provision of a well-structured list of terms that are needed to describe a specific domain. Most ontology development efforts have required not only the participation of ontology engineers but also of domain experts. However, the quality of ontologies varies widely due to absent integration of one or more of such expert competencies [6]. Different kinds of quality indicators of ontologies are affected: structural, logical, correct representation of domain knowledge and coverage.

As engineering discipline, the ontology development requires the existence and application of methodologies and standards. In the last decades, different processes, strategies, and ontology development methods have been proposed. Among the methods proposed in the nineties we can highlight TOVE (TOronto Virtual Enterprise) [7], the Enterprise Model Approach [8], or Methontology [9]. More recently, approaches based on best practices like Ontology Design Patterns (http://ontologydesignpatterns.org), principles like the OBO Principles (http://www.obofoundry.org/crit.shtml) or guidelines like GoodOD [10] have been proposed.

Apart from the availability of development methodologies, measuring the quality of the resulting ontologies is necessary in order to monitor to which extent and how good methodologies, practices and guidelines are being applied. Consequently, methods for ontology evaluation have been developed by the ontology community, which can, roughly, be divided into three groups: ontology ranking [11]–[13], formal correctness of the ontology contents [14]–[17]; and ontology quality frameworks [18]–[22].

Despite the differences between these groups, many of the approaches share some evaluation instruments like the use of quality models or metrics. Some methods use a gold standard to which the ontology is compared using ontology similarity metrics [23]–[25]. Gold standards are an important resource, but they are rarely available, especially for those domains for which new ontologies are developed.

Another evaluation approach is the use of competency questions, which can be understood as the formulation of ontology requirements as questions, for which correct answers are created by domain experts. A good ontology has to provide the correct answers to these questions with the help of automated reasoning [26]. This technique has also been mentioned by ontology evaluation [27] and construction [28] approaches. The main reason for this is that ontology development and evaluation methods are related. The final report of the Ontology Summit 2013 [29] proposes a method for developing and evaluating ontologies. The method focuses on the requirements traceability from development life cycle to quality evaluation. Five ontology quality characteristics were identified and combined in a model for ontology life cycle [29]: Requirements Development, Ontological Analysis, System Design, Ontology Development, System Development and Integration. For each phase of the model, a set of ontology evaluation criteria in terms of competency questions were defined.

Hence, it can be said that the alignment of ontology construction and evaluation methods is a current challenge that would be an important contribution to the progress in ontology engineering, although there is *only little empirical evidence* for the effectiveness of this approach. Besides, to our knowledge there are no empirical studies that compare different ontology evaluation methods. In this work we will try to make contributions to these areas by analyzing and comparing the results achieved with the GoodOD ontology construction approach [10], which promotes an ontology construction guideline and has been applied to ontology building tasks under lab conditions. We perform the analysis within the ontology evaluation framework OQuaRE and compare the results with the outcome of previous analyses of the GoodOD method [30], [31].

The ontology quality framework OQuaRE [21] addresses the traceability between requirements and metrics. Ontology requirements are traced to quality characteristics, which in turn are associated with quality subcharacteristics. Each subcharacteristic is measured through a set of metrics. At the end, each (sub)characteristic is given a score in the range 1 (worst) to 5 (best). OQuaRE has demonstrated its usefulness for evaluating existing biomedical ontologies [21], [32], but its usefulness for evaluating ontologies that had been specifically developed following good practices has not been studied so far.

The GoodOD [10] guideline for good ontology design focuses on ontology engineering principles in the spirit of the new reference discipline “Applied Ontology”, and methodological principles. This guideline provides a basic understanding required by a user with limited knowledge in logics, computer science or philosophy “to be able to solve ontology engineering problems in a foreseeable and non-arbitrary way” (http://purl.org/goodod/guideline). The effectiveness of the guideline was evaluated in a randomized controlled trial.

The objectives of this paper are (1) to employ the metrics of the OQuaRE framework as outcome parameter for the evaluation of the result ontologies derived from the GoodOD randomized controlled trial, and (2) to specify whether OQuaRE is able to provide additional useful information in addition to similarity and competency question based metrics.

## Methods

### The OQuaRE quality model

Ontology quality evaluation and Requirements (OQuaRE) is a framework for Ontology Quality Evaluation, based on the software product quality ISO 25000:2005 named as SQuaRE. The OQuaRE framework defines all the elements required for ontology evaluation: evaluation support, evaluation process and metrics. OQuaRE combines a quality model and quality metrics. It also addresses the traceability between ontology requirements and metrics.

Currently, OQuaRE includes the quality model which defines a set of quality characteristics such as structural, reliability, operability, maintainability, compatibility, transferability and functional adequacy, for evaluating ontologies. Such characteristics have been adapted from SQuaRE to ontologies except for the structural one, which has been included due to its importance in measuring ontology quality, according to requirements, principles and characteristics of ontologies. Every quality characteristic has a set of quality subcharacteristics associated, which are measured by quality metrics.

**Figure 1:**
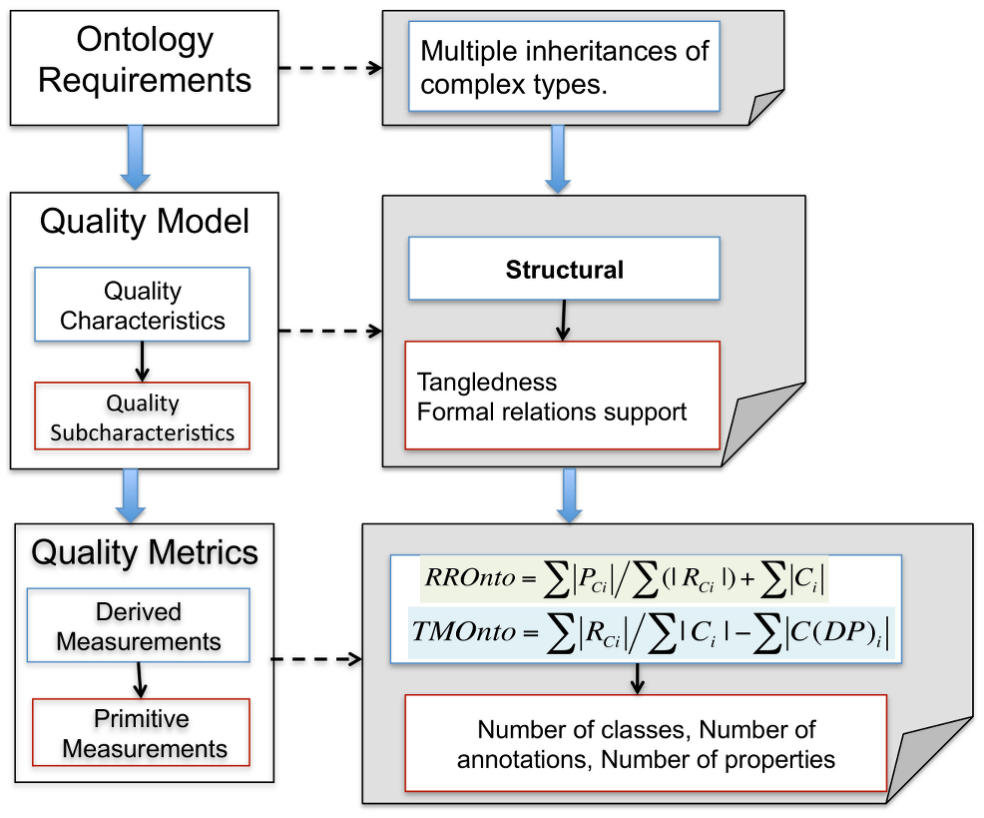
OQuaRE framework. The left side shows the components of the OQuaRE framework (Ontology Requirements, Quality Model and Quality Metrics) and the right side presents an example of traceability from ontology requeriments to metrics in OQuaRE.

The ontology requirements as such are not defined in OQuaRE but they could be traced to the Quality Characteristics which are presented in terms of the capability of the ontology to fulfill some criteria. Figure 1 shows the OQuaRE model with an example of the traceability from Ontology Requirements to Metrics.

The complete description of the OQuaRE quality model is available at http://miuras.inf.um.es/oquarewiki. The most important characteristics for this study (Structural, Functional Adequacy, Compatibility, Operability, Reliability and Maintainability) and some of its subcharacteristics are described next:

STRUCTURAL: Formal and semantic important ontological properties that are widely used in state of-the-art evaluation approaches. Some subcharacteristics are:

• Cohesion: The degree of relation between the classes. An ontology has a high cohesion if the classes are strongly related.

• Redundancy: Capability of the ontology to be informative.

• Formal relations support: Capability of the ontology to represent relations supported by formal theories different from the formal support for taxonomy.

• Tangledness: This measures the distribution of multiple parent categories, so that it is related to the existence of multiple inheritance.

FUNCTIONAL ADEQUACY: The capability of the ontologies to be deployed fulfilling functional requirements. Some subcharacteristics are:

• Controlled vocabulary: Capability of the ontology to avoid heterogeneity of the terms.

• Consistent search and query: The degree to which the formal model and structure of the ontology provides a semantic context to determine the result set wanted by the users, allowing better querying and method searching.

• Knowledge acquisition and representation: Capability of the Ontology to represent the knowledge acquired (ability to support a knowledge base of ontology individuals).

• Knowledge reuse: The degree to which the content of the ontology can be used to build other ontologies.

COMPATIBILITY: The capability of two or more software components to exchange information and/or to perform their required functions while sharing the same hardware or software environment. Some subcharacteristics are:

• Replaceability: The degree to which the ontology can be used in place of another specified ontology for the same purpose in the same environment.

• Adaptability: The degree to which the ontology can be adapted for different specified environments (languages, expressivity levels) without applying actions or means other than those provided for this purpose for the ontology considered.

OPERABILITY: Effort needed for the application. Individual assessment of such application by a stated or implied set of users. Some subcharacteristics are:

• Learnability: The degree to which the ontology enables users to learn its application.

RELIABILITY: Capability of an ontology to maintain its level of performance under stated conditions for a given period of time.

• Availability: The degree to which an ontology, or part of it, is operational and available when required for use with different applications.

• Recoverability: The degree to which the ontology can re-establish a specified level of performance and recover the data directly affected in the case of a failure.

MAINTAINABILITY: Capability of ontologies to be modified for changes in environments, in requirements or in functional specifications. Some subcharacteristics are:

• Modularity: The degree to which the ontology is composed of discrete components such that a change to one component has minimal impact on other components.

• Reusability: The degree to which an asset (part of) the ontology can be used in more than one ontology, or in building other assets.

• Analysability: The degree to which the ontology can be diagnosed for deficiencies or causes of failures (inconsistences), or for the parts to be modified to be identified.

• Changeability: The degree to which the ontology enables a specified modification to be implemented. The ease with which an ontology can be modified.

• Modification stability: The degree to which the ontology can avoid unexpected effects from modifications of the knowledge (terms, classes, properties, etc.).

• Testability: The degree to which the modified ontology can be validated.

#### OQuaRE quality metrics

Quality metrics are composed of primitive and derived measurements. Primitive measurements correspond to metrics that can be measured directly on the ontology, such as number of classes, number of relations, etc, whereas derived ones combine some primitive measurements. A set of quality metrics has been reused and adapted from both ontology and software engineering communities. Some metrics adapted from software engineering are presented in Table 1 (all abbreviations introduced there): RFCOnto, NACOnto, NOCOnto [33], [34], and some metrics adapted from the ontology community are LCOMOnto, RROnto, AROnto, CROnto [13], [35].

**Table 1:** OQuaRE Metrics.

**LCOMOnto** (Lack of Cohesion in Methods): Length of the path from the leaf class to Thing, divided by the total number of paths in the ontology
**WMCOnto** (Weighted Method Count): Average number of Datatype Properties, Object Properties and subclasses per class
**DITOnto** (Depth of Inheritance Tree): Length of the largest path from Thing to a leaf class of the ontology
**NACOnto** (Number of Ancestor Classes): Average number of superclasses per leaf class
**NOCOnto** (Number of Children): Average number of the direct superclasses per class minus the subclasses of Thing
**RFCOnto** (Response for a Class): Number of Datatype Properties and Object Properties that can be directly accessed from the class
**NOMOnto** (Number of Properties): Average number of Datatype Properties and Object Properties per class
**PROnto** (Property Richness): Ratio of the number of Datatype Properties and Object Properties defined in the ontology divided by the number of subclasses, Datatype Properties and Object Properties
**AROnto** (Attribute Richness): Number of restrictions of the ontology divided by the number of classes
**INROnto** (Relationships per Class): Average number of subclasses per class
**CROnto** (Inheritance Relationships Richness): Average number of individuals per class
**ANOnto** (Annotation Richness): Average number of annotation properties per class

Definition of the metrics used in OQuaRE. In the definition of the metrics, number refers to assertions in the ontology and not to the number of entities defined in the ontology.

The evaluation of an ontology in the OQuaRE framework comprises a score for each quality characteristic, which depends on the evaluation of the set of associated subcharacteristics. The evaluation of a particular subcharacteristic depends on its associated metrics.

One metric can be associated with one or more subcharacteristics, and therefore may contribute to the score of more than one subcharacteristic. For instance, ANOnto “Annotation properties per class” contributes to knowledge reuse (of functional adequacy) and Redundancy (of structural), because annotation properties represent additional knowledge useful for reusing, so it is more informative and helps to be less redundant. Consequently, ANOnto contributes to Structural and Functional Adequacy characteristics.

The metrics are presented in different scales. For instance, RFCOnto and ANOnto generate absolute values whereas CROnto and INROnto produce relative values. Hence, in order to measure a quality subcharacteristic, those values are mapped onto the range 1 to 5. This mapping is based on the SQUaRE scores of the quality characteristics and subcharacteristics where values are ranged between 1 and 5. A value of 1 means “not acceptable”, 3 is “minimally acceptable”, and 5 is “exceeds the requirements” [36].

It should be noted that high values in the metrics might not correspond to a high quality score. For this purpose we apply the recommendations and best practices of the Software Engineering community for software metrics and ontology evaluation metrics.

Finally, the score of each subcharacteristic is calculated by the weighted average of the scores for its metrics. Likewise, the score of each characteristic is calculated by the weighted average of the scores of its subcharacteristics. The scoring mechanism based on weights provides a flexibility which permits to adapt the method to the need of a particular community.

### The Good Ontology Design (GoodOD) Guideline

In the Good Ontology Design (GoodOD) project a guideline was formulated describing principles of good ontology design (http://purl.org/goodod/guideline). The main objective of this guideline is to help domain experts to develop good ontologies focusing on good practices. The GoodOD guideline brings together good practice rules, ontology design patterns and top-level categories, in order to provide ontology developers with standard solutions to recurrent modelling tasks.

The guideline comprises information on the definition of the basic elements of an ontology, its taxonomic structure, the formal language used to develop ontologies, and description logics (DL). The most important classes and relations from the upper domain ontology BioTop [3] are introduced to provide a framework for practical modeling tasks from the biomedical domain. This guideline combines foundations from philosophical ontology and logics, with the representational language OWL DL, and is rooted in the domain top-level ontology BioTop [10].

The effectiveness of the GoodOD guideline was evaluated in a randomized controlled trial in which the performance of ontology developers was compared after specific and unspecific training. As outcome parameters gold-standard based similarity measures [30] and a competency question based approach [31] were adopted. The study is described in more detail in the following sections.

Main results and conclusions from the study were that in the gold-standard based evaluation approach no significant effect of the specific training on the quality of ontologies could be detected. However, these results are not sufficient to conclude that there are generally no effects of the GoodOD principles. With the competency-question based approach a small but significant effect of the guideline-based training over the unspecific training could be shown (about 10% better). However, when analyzed on the level of single topics, a significant effect was measureable only for one topic.

Due to the high complexity and the educational nature of the study, the results should be carefully interpreted. The small effects of this study do not mean that construction principles should be abolished. They indicate that better evaluation methods must be developed. It is also optimistic to expect that significant training effects can be measured already after a one-week training phase. To our knowledge, the GoodOD study is the only empirical study investigating the effects of construction principles and ontology development guidelines on the quality of resulting ontologies so far.

### Study Design

In this study the results of a randomized controlled trial are re-assessed by a complementary ontology evaluation framework (OQuaRE) and compared with prior results.

### Randomized controlled trial on the effectiveness of guideline-based ontology training

The objective of this trial was to investigate whether guideline-based ontology training has an effect on the performance of ontology developers. The intervention of this trial was a curriculum implementing the principles of the GoodOD-guideline.

DESIGN: The study was conducted as a randomized controlled trial with a crossed intervention in a post-test only design.

POPULATION AND SAMPLE: The target population of the study were students with a combination of subjects from the Life Sciences and Computer Sciences. 24 students with a proven combination of the subjects in Biology or the Life Sciences and Computer Science from Austria, Germany, Slovenia and Switzerland were recruited. The study was conducted in summer 2011 at the Department of Medical Biometry and Medical Informatics of the University of Freiburg, Germany.

ALLOCATION: The students were randomized and allocated to one of two intervention groups.

INTERVENTION: A curriculum implementing the GoodOD-guideline (see above) in 16 modules was used as intervention. After a 2.5 days training in which both groups received the same training, the groups were randomized and received differential training for 1.5 days.

In the differential training phase, modules with different content but balanced in difficulty and duration were taught to each group (see Table 2). Group A was trained on the subject-matters *Process and Participation* (PRO), *Immaterial object* (IMM) and the *Closure* ODP (CLO), whereas group B was trained on *Collective material entity* (CME), *Information object* (INF) and the *Spatial disjointness* ODP (SPA).

**Table 2:** Intervention of the study, ontology topics and modules.

Module type	Topic	Group Trained
Top-level categories	Process and Participation (PRO)	A
	Immaterial object (IMM)	A
	Collective material entity (CME)	B
	Information object (INF)	B
Ontology design patterns	Closure ODP (CLO)	A
	Spatial disjointness ODP (SPA)	B

OUTCOME PARAMETERS AND DATA COLLECTION: After the training phase, the students were asked to fulfill 12 modeling tasks with different content. Each student developed 12 test ontologies, six ontologies for the three topics in which they had received specific guideline based training and six ontologies for the three topics in which they only received unspecific training. It should be noted that the assessment of each topic included two different ontology development tasks. The students developed the test ontologies with the Protégé ontology editor, so that 12 OWL ontologies were collected from each student in 12 individual OWL files resulting in a total of 288 files.

The outcome parameter of the study was the quality of the resulting ontologies. The main outcome parameter was the quality of the ontologies measured as the similarity with gold standard ontologies and the secondary outcome parameter was the quality of the ontologies measured with a competency-question based approach.

In this work, OQuaRE is introduced as a new set of secondary outcome parameters. The authors of the GoodOD study see this extension of outcome parameters, although not declared in the original study protocol, as a legitimate and valuable application of further quality measures on the original research question of the study. For the detailed application of OQuaRE see the next section.

ANALYSIS AND REPORTING: For the calculation of similarity between students' ontologies and gold standard ontologies, a set of gold standard ontologies was developed by the authors of the GoodOD study. A software, the Good Similarity Evaluator [37], automated the process of similarity calculation on the basis of prior work [23], [25], [38], [39].

For the competency-question based measurement a set of questions and their allowed answers were designed by the experts. They were expressed in Manchester OWL 2 Syntax as DL axioms. The competency questions were used to check different aspects of the ontologies, such as the correctness of the asserted hierarchy, the correct and exhaustive introduction of disjointness axioms, the correct usage of classes and relations of top-level ontologies and the correct usage of ODPs.

The reporting of the study follows the CONSORT statement for the reporting of randomized controlled trials [40].

ETHICAL APPROVAL: Ethical approval was requested from the ethical authority of the University of Freiburg, Freiburg, Germany. The chair of the University of Freiburg ethics committee reviewed the project and concluded that a full formal ethics committee statement was not required due to the educational nature of the study. It was designed according to the general requirements for educational studies at the University Medical Center Freiburg, Freiburg, Germany, and was performed with written informed consent of the participants.

### Application of OQuaRE as an outcome parameter of the GoodOD study

In this experiment, 288 OWL files corresponding to 12 ontologies developed by 24 students were reused. Each ontology was evaluated using a predetermined OQuaRE configuration that will make use of 29 OQuaRE subcharacteristics. The application of OQuaRE was done using a home-made Java tool, which calculates the corresponding scores and returns a score in the range 1 to 5 for each characteristic, subcharacteristic and metric.

A random selection of ontologies was performed to obtain independent ontology samples with equal sample sizes in each of the 12 combinations of untrained and trained students by topic, balancing the inability of the tool to process all of the 288 OWL files; 6 randomly selected OWL files per ontology from different students were discarded from the study. The process assured that the data contained ontologies from all of the 24 students. Consequently, the final set of data consisted of 216 OWL files corresponding to 12 ontology construction tasks, each one tackled by 18 different students (9 trained, 9 untrained) randomly selected from a set of 24 students, and supplemented by two gold standard ontologies.

Next, we describe the methodological approach followed to perform the statistical analysis of the data which was done using R packages. Firstly, using data from the evaluation of students' ontologies, a two-way ANOVA model [41] with balanced independent samples design was used to identify the effect of the main factor training by topic in each of the 29 OQuaRE subcharacteristics. The interaction mean plot allowed to visualize the effect of the training along the topics and, in the situation where the interaction term was significant, the Student's t-test was performed to analyse the differences between the means of untrained and trained groups.

Secondly, to compare the gold standard against the students, the mean distances from both untrained and trained students to the gold standard were used. This distance was computed by means of the difference in absolute value of the gold standard measure minus the untrained and trained students observed scores, respectively.

Additionally, descriptive multivariate analysis was used to analyse the 29 OQuaRE subcharacteristics together. Clustering methods were applied to describe, through internally homogeneous groups of subcharacteristics, the change of those groups from untrained to trained case. Principal Component Analysis was applied to quantify and describe in detail this change.

## Results

In this section we present the main results of our data analysis. For the sake of simplicity, we do not show the OQuaRE scores for all of the ontologies, which can be accessed at http://miuras.inf.um.es/oquarewiki.

### Analysis of the effect of the GoodOD guideline over the quality of the ontologies developed

**Figure 2:**
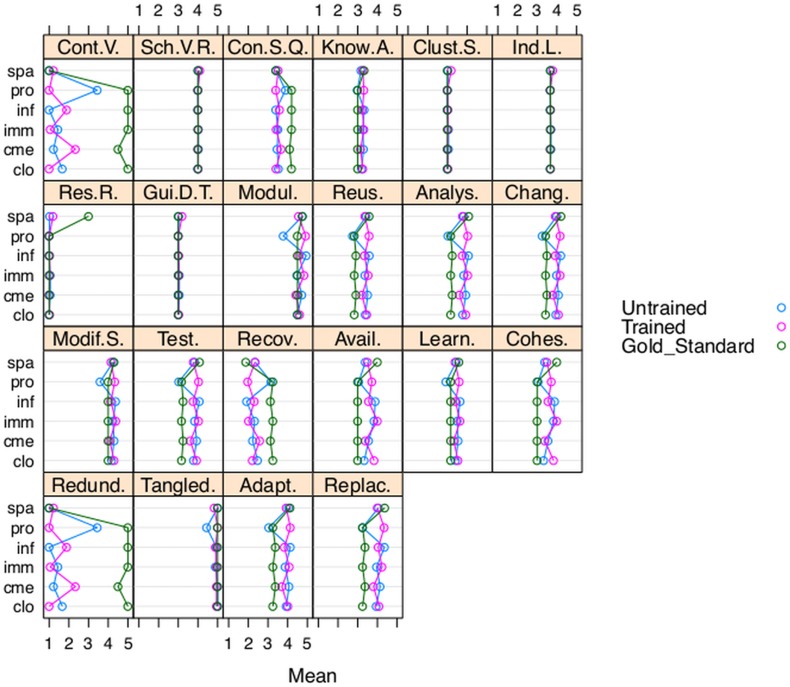
Significant effect of training in some topics. 22 subcharacteristics presented significant effect due to the GoodOD based training for some topics (PRO, IMM, CLO, CME, INF, SPA).

**Figure 3:**
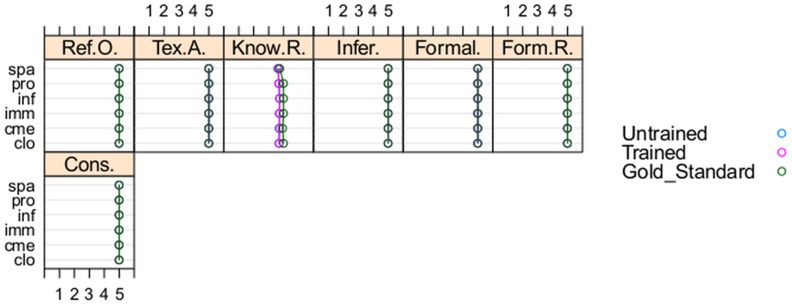
No significant effect of training in any topic. Seven OQuaRE subcharacteristics presented no significant effect of the training for any topic (PRO, IMM, CLO, CME, INF, SPA), and their mean values were similar for students and the gold standard.

To analyse the training effect over the ontologies developed by the students, a training by topic two-way ANOVA model with interaction term and balanced design for everyone of the 29 evaluated subcharacteristics was applied. The 216 observations from 24 untrained and trained students, corresponding to 36 ontologies for each of the six topics (PRO, CLO, IMM, INF, CME and SPA) were reused. This analysis showed that there is a significant effect of the interaction training by topic for 22 OQuaRE subcharacteristics (.000<*p–value*<0.03) (Figure 2). This means that the effect of the training could be different depending on the topic for those 22 selected subcharacteristics. For the other seven subcharacteristics there is no effect of the interaction training by topic, neither by topic neither by training (see Table 3 and Figure 3).

**Table 3:** P-values associated with the main effects in the two-way ANOVA with no effect of the interaction training by topic.

Sub-characteristic	Trained	Topic	Trained X Topic
Reference Ontology	0.318	0.418	0.418
Text Analysis	0.318	0.418	0.418
Knowledge Reuse	0.586	0.069	0.952
Infering	0.318	0.418	0.418
Formalisation	0.318	0.418	0.418
Formal Relation Support	0.318	0.418	0.418
Consistency	0.318	0.418	0.418

Figure 3 represents the mean values of the subcharacteristics by topic, for untrained students, trained students and the gold standard. The graph shows that the seven subcharacteristics with no significant effect of the training for any topic presented similar mean values for students and gold standard.

For the 22 subcharacteristics with significant interaction effect (see Figure 2), new analysis to identify the main effect of the training were done. For those analyses, the topic factor was fixed and Student's t-tests were applied. The results show that there are no significant differences between untrained and trained students for topic SPA for none of the 22 subcharacteristics. Significant differences (*p–value*<0.03) were found for topics CME, IMM, CLO, INF and PRO. Concretely, IMM and CLO presented significant effect of training for one and two subcharacteristics respectively. INF presented significant effect of training for six subcharacteristics and CME presented significant effect of training for eleven subcharacteristics with significance levels less than 0.05 (0.02<*p–values*<0.05). Finally, in PRO, 17 subcharacteristics presented significant effect of training and 12 of them with a high significance level less than 0.000 (*p–values*<.000).

**Figure 4:**
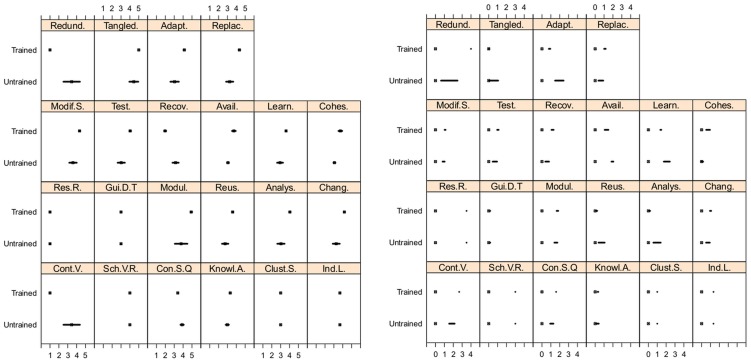
Confidence intervals for Topic PRO: for trained and untrained students (left side); distances to gold standard (right side).

The left side of Figure 4 shows the differences between untrained and trained students through the 95% confidence interval for its mean values for the 22 studied subcharacteristics in topic PRO. The untrained group shows wider intervals than the trained one, so that, the mean of the untrained group has a greater standard error than the trained one.

In summary, twelve subcharacteristics did not reveal any training effect in any of the topics (see Table 4). However, the effect of training was significant for the remaining 17 subcharacteristics for topic PRO and some of them for topics CME, INF, CLO and IMM. The effect of training was not significant for any subcharacteristics in topic SPA. These results provide support to say that the GoodOD training presented important effect over the quality of the developed ontologies for PRO and CME, medium effect for INF, low effect for CLO and IMM and no significant effect for SPA. Comparing trained against untrained students, PRO showed a positive effect (higher OQuaRE subcharacteristics score) for 12 of the 17 subcharacteristics (see Figure 2). Additionally, the training effect for PRO can be analyzed using the dispersion (see Figure 4), which is greater for untrained students than for trained ones, except for Cohesion. This shows that the set of ontologies developed by trained students is more homogeneous than the set developed by untrained ones.

**Table 4:** Significance levels of testing difference of means.

SubCharacteristic	CLO	CME	IMM	INF	PRO	SPA
Reference Ontology	- -	- -	- -	- -	- -	- -
Text Analysis	- -	- -	- -	- -	- -	- -
Infering	- -	- -	- -	- -	- -	- -
Formalisation	- -	- -	- -	-	- -	- -
Formal Relation Support	- -	- -	- -	-	- -	- -
Consistency	- -	- -	- -	- -	- -	- -
Schema And Value Reconciliation	- -	- -	- -	- -	-	- -
Indexing And Linking	- -	- -	- -	- -	- -	- -
Clustering And Similarity	- -	- -	- -	- -	- -	- -
Guidance And Decision Trees	- -	- -	- -	- -	- -	- -
Results Representation	- -	- -	- -	- -	- -	- -
Knowledge Reuse	- -	- -	- -	- -	- -	- -
Tangledness	- [***]	- -	- -	- -	(*)[*]	- -
Modularity	- -	- -	- -	- -	(**)	- -
Knowledge Acquisition	- -	- [*]	- [*]	- [*]	(**)	- -
Modification Stability	- [*]	- -	- -	- -	(**)[*]	- -
Learnability	- -	- -	- -	- -	(**)[**]	- -
Reusability	- -	- -	- -	- -	(***)[**]	- -
Changeability	- -	(*) -	- -	- -	(***)[*]	- -
Availability	(**)[**]	(*) -	- -	(*)[*]	(***)[***]	- -
Cohesion	(**)[**]	(*) -	- -	(*)[*]	(***)[***]	- -
Analysability	- -	(*) -	- -	- [*]	(***)[**]	- -
Testability	- -	(*) -	- -	- -	(***)[*]	- -
Adaptability	- -	(*)[***]	- -	- -	(***)[***]	- -
Consistent Search And Query	- -	(*)[*]	- -	(*)[*]	(***)[***]	- -
Replaceability	- -	(*)[***]	- -	- -	(***)[**]	- -
Recoverability	- -	(*) -	(*)[*]	(*) -	(***)[***]	- -
Controlled Vocabulary	- -	(*)[*]	- -	(*)[*]	(***)[***]	- -
Redundancy	- -	(*)[*]	- -	(*)[*]	(***)[***]	- -
**Total Significant differences**	(2)[4]	(11)[7]	(1)[2]	(6)[7]	(17)[15]	(0)[0]

Differences between means of untrained and trained groups () and mean distances to the gold standard of trained and untrained groups []: The character * is used to indicate the significance level of the differences: * significant, ** very significant and *** highly significant.

### Analysis of the distances between the students and the gold standard

The mean distances from both, untrained and trained students to the gold standard were obtained and the difference analyzed for every topic (PRO, CME, CLO, IMM, INF and SPA). In each case, the distance was computed by means of the difference in absolute value of the gold standard measure minus the observed score from students.

As previously mentioned, Figures 3 and 2 also represent the mean values of the subcharacteristics for the gold standard ontologies and Figure 3 showed that the gold standard and students had similar behaviour for seven subcharacteristics in all of the topics. In fact, for those subcharacteristics, there are no significant differences between the mean of the distances from untrained and trained students to the gold standard in any topic. For the remaining 22 subcharacteristics (see Figure 2) the analyzed differences provide enough evidence that training had significant effect in some subcharacteristics for CLO, CME, INF, IMM and PRO; but none for SPA. For PRO, significant differences between the mean distances from untrained and trained students to the gold standard were found for 15 subcharacteristics; confidence intervals are shown in the right side of Figure 4.

The effect of the training is summarized in Table 4, where ( ) and [ ] represent the significance level of the difference between the means of untrained and trained students, and between the means distances from untrained and trained students to the gold standard, respectively. The last row of the table shows the total number of significant differences. The highest effect of the training was found for PRO, followed by CME and INF which presented the second and third highest effect respectively.

In conclusion, the effect of the training was significant in 17 subcharacteristics, either due to differences between untrained and trained means, or between untrained and trained mean distances to the gold standard. We consider that this effect is high for PRO, intermediate for CME and INF, and small for CLO, IMM, and no effect was found for SPA.

### Analysis of quality of the ontologies by classifying and sorting the sub-characteristics

We have performed a multivariate analysis of the means distances from untrained and trained students to the gold standard in the set of the six topics (CLO, CME, IMM, INF, PRO, SPA), to describe the 29 OQuaRE subcharacteristics together. First, the original data were transformed by equation 1, where *x*_1_, *x*_2_ and *G* represent the mean values of the ontologies from untrained students, trained students and the gold standard ontologies respectively.
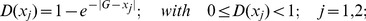
(1)


This transformation was applied to all of the subcharacteristics for the six topics, obtaining two 29*x*6 data matrices, namely, *D*_1_ and *D*_2_. These matrices correspond to the mean distances from untrained and trained students to the gold standard respectively. Classification and sort methods were applied to the matrices to identify similar behaviours between subcharacteristics for both untrained and trained cases with respect to their distance to the gold standard.

**Figure 5:**
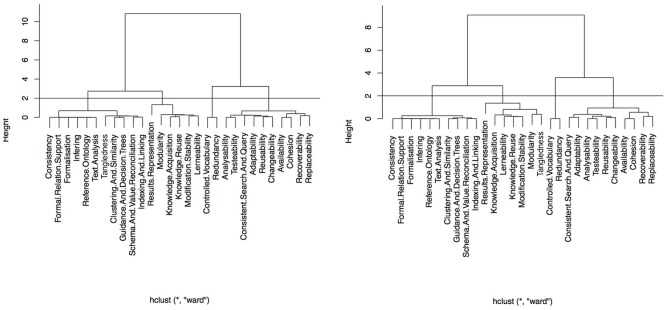
Subcharacteristics classification for untrained and trained students, respectively.

CLASSIFYING SUBCHARACTERISTICS. Based on the Euclidean distance between subcharacteristics and Ward's minimum variance method of linkage criteria, hierarchical clustering was applied for both cases. The hierarchical cluster allowed to classify the subcharacteristics in four homogeneous groups for untrained and trained cases (see Table 5 and Figure 5).

**Table 5:** Cluster centers.

			UNTRAINED GROUP			
Group	CLO	CME	IMM	INF	PRO	SPA
1	0.00	0.02	0.02	0.00	0.00	0.01
2	0.96	0.96	0.97	0.98	0.79	0.00
3	0.43	0.48	0.50	0.57	0.12	0.24
4	0.12	0.18	0.16	0.23	0.25	0.19

			TRAINED GROUP			
Group	CLO	CME	IMM	INF	PRO	SPA
1	0.01	0.01	0.01	0.02	0.00	0.07
2	0.98	0.89	0.98	0.96	0.98	0.20
3	0.54	0.32	0.59	0.42	0.58	0.28
4	0.20	0.10	0.25	0.15	0.25	0.25

• Group 1 includes ten subcharacteristics for the untrained students plus Tangledness for the trained ones. The means distances from untrained and trained students to the gold standard are low for the six topics.

• Group 2 includes only two subcharacteristics for both untrained and trained students. The distances to the gold standard were high for all topics except for SPA, which were zero for the untrained students and low for the trained ones.

• Group 3 includes ten subcharacteristics in both untrained and trained students. The distances to the gold standard decrease from untrained to trained in CME and INF but increase for the other four topics, the highest one being found for PRO.

• Group 4 includes the same seven subcharacteristics for both untrained and trained cases, except for Tangledness which appears in group 1 for the trained students. This group presented the same behaviour as group 3 but with shorter distances for both trained and untrained students for all of the topics, except for PRO which presented the same value for both untrained and trained students.

In summary, the classification of subcharacteristics were the same along the topics, except for Tangledness which changed from group four in untrained case to group one in trained one, the distances to gold standard being larger for untrained students than for trained ones.

Additionally, a K-means classification with four groups obtained the same distribution of subcharacteristics as hierarchical cluster except for *Results Representation* and *Tangledness*. In contrast to hierarchical clustering, they were classified in group one for both untrained and trained students. The quality of this classification was measured by the percentage of total variance explained (between groups) which was 93% for untrained students and 87% for trained ones. The unexplained variance (within groups) was 4.9%, 0.0%, 1.9% and 0.6% for untrained students and 5.6%, 0.0%, 3.5% and 3.4% for trained ones, respectively in each group.

**Figure 6:**
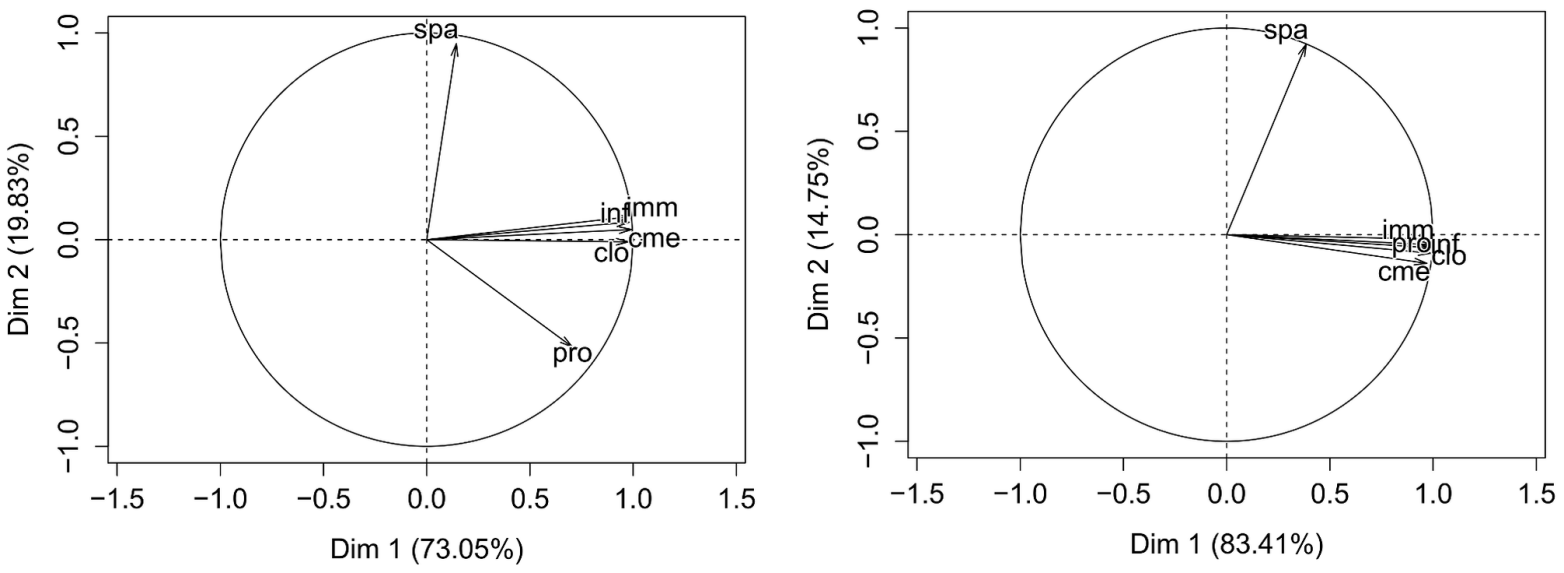
Factor loadings of topics in Principal Component Analysis. Untrained case (left) and trained case (right). With the two more important components, more than 98% of total variance was explained for untrained students, and more than 92% of total variance for trained ones.

SORTING SUBCHARACTERISTICS. Principal Component Analysis (PCA) was applied to order the subcharacteristics by distance between untrained or trained groups to the gold standard for every topic. PCA was applied to the *D*_1_ and *D*_2_ matrices to represent the subcharacteristics in two dimensions. The representations obtained were of high quality: more than 98% of total variance was explained for untrained students, and more than 92% of total variance for trained ones, as is shown in Figure 6. This figure also represents the correlations between the topics and the principal components for both cases.

The first component was highly correlated with CLO, CME, IMM, INF and PRO for both untrained and trained students. Therefore, subcharacteristics with highest positive values in this component presented the highest mean distances to the gold standard for those topics.

The second component was highly and positively correlated with SPA for both untrained and trained cases and highly negatively correlated with PRO for untrained case. Consequently, subcharacteristics with high positive values in this component presented the largest differences in distance to the gold standard, for both untrained and trained cases for SPA. Additionally, for the untrained case, subcharacteristics with high negative values in this second component presented the largest distances to the gold standard for PRO.

Values for first and second principal components and membership clusters are displayed in Table 6, where subcharacteristics are ordered by the first component for the untrained cases (PC1_U).

**Table 6:** Sorting and classification of the 29 OQuaRE subcharacteristics for untrained (U) and trained (T) cases, respectively.

Subcharacteristics	PC1 U	PC2 U	Cluster U	PC1 T	PC2 T	Cluster T
Text Analysis	−2.1415	−0.4745	1	−2.3298	−0.7121	1
Infering	−2.1415	−0.4745	1	−2.3298	−0.7121	1
Formalisation	−2.1415	−0.4745	1	−2.3298	−0.7121	1
Formal Relation Support	−2.1415	−0.4745	1	−2.3298	−0.7121	1
Consistency	−2.1415	−0.4745	1	−2.3298	−0.7121	1
Reference Ontology	−2.1415	−0.4745	1	−2.3298	−0.7121	1
Schema And Value Reconciliation	−2.0443	−0.3984	1	−2.1722	−0.1843	1
Indexing And Linking	−2.0099	−0.3925	1	−2.1285	−0.0359	1
Clustering And Similarity	−1.9497	−0.3236	1	−2.0242	0.2940	1
Guidance And Decision Trees	−1.9497	−0.3236	1	−2.0242	0.2940	1
Results Representation	−1.6551	3.4082	4	−1.3535	4.1078	4
Tangledness	−1.2330	−1.3450	4	−1.7404	0.0911	1
Knowledge Acquisition	−0.5027	0.3478	4	−0.5424	−0.5982	4
Modularity	−0.4209	−1.4542	4	−0.8249	0.1186	4
Knowledge Reuse	−0.1599	−0.2951	4	−0.4159	−0.5302	4
Modification Stability	−0.0551	−0.8153	4	−0.5887	−0.1009	4
Learnability	0.1805	−0.1507	4	−0.0425	−0.0061	4
Reusability	0.9643	0.2956	3	1.1085	−0.1203	3
Changeability	1.0905	0.3954	3	1.1980	0.2042	3
Availability	1.1399	1.8666	3	1.8513	0.9520	3
Cohesion	1.1399	1.8666	3	1.8513	0.9520	3
Analysability	1.4098	0.6158	3	1.6183	0.2622	3
Testability	1.4098	0.6158	3	1.6183	0.2622	3
Adaptability	1.4423	−0.1577	3	1.4247	−0.2628	3
Replaceability	1.5039	1.1593	3	2.1282	0.4131	3
Consistent Search And Query	1.5533	−0.6265	3	1.5650	−0.6867	3
Recoverability	2.1280	1.4822	3	2.7585	0.6801	3
Redundancy	5.4335	−1.4619	2	5.3570	−0.9166	2
Controlled Vocabulary	5.4335	−1.4619	2	5.3570	−0.9166	2

The first component of the untrained case is significantly correlated with the first one of the trained case, consequently, a relation between behaviours for both cases was observed for CLO, CME, IMM, INF and PRO:

• Redundancy and Controlled Vocabulary obtained positive and atypical values in the first component, which means that distances to the gold standard were high in both cases.

• Tangledness and Modification stability presented the largest decrease of mean distances to the gold standard from untrained to trained cases. Conversely, Recoverability, Cohesion, Availability and Replaceability presented the highest increase in distance to the gold standard from untrained to trained case.

The second component showed that:

• Result Representation obtained highest and atypical values in both untrained and trained cases, and the distances to the gold standard were high for SPA.

• Tangledness, Modification Stability, Result Representation, Modularity, Clustering and Similarity, Guidance and Decision Trees had the highest increase of the mean distances to the gold standard from untrained to trained cases. Conversely, Knowledge Acquisition, Availability, Cohesion, Replaceability and Recoverability presented the highest decrease from untrained to trained case.

In conclusion, the first principal component sorting showed that PRO, IMM, CLO, CME, and INF are highly correlated. The mean distance for these topics decreased from the untrained cases to the trained cases for 19 subcharacteristics, most of them with negative values in both cases (this means that mean distance are lower than the general mean). In contrast, this mean distance increased from untrained to trained for the other 10 subcharacteristics, and most of them presented positive larger distance to the gold standard (this means that mean distance is larger than the general mean), except for Result Representation, for which distances were negative.

The second component presented a high correlation with SPA for both untrained and trained cases, and with PRO for the untrained ones. The first and second components are uncorrelated, therefore, the behaviour of SPA is independent of the behaviour of the rest of topics in both untrained and trained cases.

## Discussion

In this study, OQuaRE was used to evaluate a set of biomedical ontologies developed by 24 students after guideline-based training. To measure the effect of the training over the properties of the ontologies, two different studies were carried out: (1) comparative analysis of the properties of the ontologies developed by untrained and trained students; (2) analysis of the distances of both sets of ontologies with respect to a set of gold standard ontologies. Additionally, the subcharacteristics have been ordered and classified according to the respective distances to the gold standard solutions.

### Comparison with previous evaluations of the GoodOD guideline

Our research objectives were inspired by the work presented in [30], [31]. Consequently we compare the present results with these two studies. We first analyzed the effect of training over the quality of the ontologies. In [31], the evaluation was based on competency questions metrics and found an effect of training for two topics, PRO and CME, and the differences were significant only for the first one. In our study, we have analyzed the effect of the training for 22 subcharacteristics, where the effect of topic by training interaction was significant. The effect was highly significant for six subcharacteristics in INF, 11 in CME and 17 in PRO. Additionally, in this last topic, ontologies developed by trained students were more homogeneous than the set developed by untrained ones. Hence, both studies draw similar conclusions.

Second, we have tested whether the ontologies developed by trained students were closer to the gold standard than those developed by the untrained ones were. In [30], a set of similarity measures of the ontology artifacts revealed that the ontologies developed by trained students were not significantly more similar to the gold standard than those developed by untrained students. Our findings reveal that there is a significant effect of training with respect to the gold standard (Table 4), in both negative and positive directions for PRO, IMM, CLO, CME and INF. SPA did not present significant differences from trained neither untrained students to the gold standard. Additionally, the classification and sorting of subcharacteristics showed that students reached values more similar to the gold standard after training with respect to 19 subcharacteristics, whereas their results were less similar to gold standard after training with respect to the 10 other subcharacteristics (see Table 6). Such conclusions were drawn for PRO, IMM, CLO, CME and INF, which are highly correlated in pairs and with the first component (see Figure 6). On the other hand, SPA is uncorrelated with the rest of the topics, behaving differently for both untrained and trained cases. After training, 18 subcharacteristics reached in SPA values more similar to the gold standard and 11 were less similar.

Consequently, we believe that these results permit to say that OQuaRE is able to capture the findings of the previous GoodOD evaluations, which was one major objective of this work.

### Findings in the ontologies produced by the students

A second objective was to study what additional information OQuaRE could provide about the quality of the ontologies developed and the effectiveness of GoodOD. According to our results, the GoodOD training had a significant effect on 59% of OQuaRE subcharacteristics and therefore on the quality of the ontologies developed by the students, and these differences were identified between ontologies developed by specific training and those without it.

Mean values for ontologies developed by untrained students and trained students, and for the gold standard ontologies were similar and high for seven subcharacteristics (see Figure 3). The highest quality scores were obtained for Tangledness for all topics except for PRO, in which Tangledness obtained a better value after the training. These subcharacteristics are associated with the general training of the GoodOD guideline, which seems to have been effective.

In general terms, Tangledness and Modification Stability obtained the highest positive effects of the training. The ontologies developed after the training presented less multiple inheritance and were more stable for changes in their classes, properties and terms. This effect might be due to the training of the GoodOD guideline, where a quality criterion is to construct ontologies in a way that multiple parenthood is inferred and not asserted.

Additionally, Recoverability, Cohesion, Availability and Replaceability obtained the most negative effects of the training. Such subcharacteristics are associated with the dependence between ontology classes.

More findings can be described when focusing on specific topics. Comparing the data for PRO in Figure 4 and Table 4, training had a significant effect on all the Maintainability subcharacteristics: Analysability, Changeability, Modification Stability, Testability and Reusability. Such differences might be induced by the training. A similar conclusion may be drawn for the rest of the subcharacteristics for which the training effect was significant. It should be noted that the most notable evidence of the effect of training is the homogeneity of the results of the trained students compared to those of the untrained students (see Figure 4).

For CME, Figure 4, shows the effect of the training on subcharacteristics such as Redundancy, Control Vocabulary, Availability, Recoverability, Adaptability, Replaceability, Learnability and Knowledge Acquisition. Thus, the scores of the ontologies from trained students are more similar to the gold standard than those from untrained students.

In Table 6, the largest difference between the gold standard and mean values for untrained and trained students was found in two subcharacteristics: (1) *Redundancy*, which is the capability of the ontology to be informative; and (2) *Controlled vocabulary*, defined as the capability of the ontology to avoid heterogeneity of the terms. Both subcharacteristics are related to the number of annotations per class. We have found that ontologies of trained students included annotations for less than 10% of the classes, whereas the gold standard included them for almost 100%. For topics SPA, INF and CME, untrained students presented lower values than trained ones for those subcharacteristics. This means that training had a positive effect with respect to those specific subcharacteristics. For topics PRO, IMM and CLO, the training effect was negative. This might be due to the annotations created by the students before and after the training. Although finding an explanation for such behaviour is out of the scope of this work, we believe that such information would provide an interesting feedback for further developments and applications of the GoodOD guideline.

These findings lead to the conclusion that OQuaRE is able to provide fine-grained information about the quality of the ontologies because of its organization in quality characteristics and subcharacteristics.

### Limitations and Future Work

As it has been aforementioned, due to technical limitations, we have not been able to work with the complete original dataset. Consequently, the study was designed to reduce potential biases. Our design was based on independent samples with equal sample sizes to use an F statistic in the ANOVA analysis less sensitive to the eventual non-compliance of the normality and homocedasticity hypotheses of the sample. In a different situation, we would have needed to test the hypotheses the ANOVA model is based on and to deal with the non-uniqueness of the solution.

The current version of OQuaRE lacks a module for the definition of a set of Ontology Quality requirements which would permit a more direct traceability between competency questions and quality metrics, allowing the measurement of the ontologies from the development phase. Additionally, we are working on including new axiom-based metrics in the framework. Besides, new experiments involving both OQuaRE and GoodOD would permit a coordinated evolution of approaches for guiding the construction of the ontologies and to evaluating those ontologies.

It should be noted that neither the OQuaRE metrics nor the GoodOD methods are meant to be sufficient for a full picture of the quality of an ontology, even when combined. Nevertheless, the work that we present in this paper is one of the very first efforts to combine different methods and our results suggest that it is a promising way to proceed. We aim at extending our approach with new metrics and combining our results with other ontology quality approaches; a possible candidate for such an extension could be the measurement of the cognitive quality of ontologies [22], which could also be included as part of the OQuaRE measurements. This approach proposes a method for evaluating the relationship between a cognitive conceptualization and an explicit specification, but assumes that the quality of an ontology is relevant and useful only in relation to that of another ontology. Consequently, it might provide additional information for studying similarities between the gold standard and the students' ontologies.

## Conclusion

In this work, OQuaRE was used to evaluate the quality of a set of ontologies developed using the GoodOD guideline for good ontology design. The quality of the ontologies was previously measured a competency-question based approach and the measurement of the similarity to gold-standard ontologies. A comparison of OQuaRE with the two previous evaluations was done.

OQuaRE results showed a significant difference in terms of quality between untrained and trained students for three topics. Additionally, compared to the gold standard, training resulted in both positive and negative effects on five topics. OQuaRE was able to draw similar conclusions as the original GoodOD evaluations, and to provide specific results such as correlations between different topics, the set of subcharacteristics affected by training, and the differences, by subcharacteristics, among ontologies developed by untrained and trained students and gold-standard ontologies.

Summarized, GoodOD and OQuaRE can be used together to take advantage of their respective strengths to develop and evaluate ontologies. To the best of our knowledge, this is one of the very first studies that attempts to compare and combine independent ontology development and evaluation methods. We believe this to be an important contribution to the progress of the ontology engineering community.
